# Assessing the utility of whole-genome amplified serum DNA for array-based high throughput genotyping

**DOI:** 10.1186/1471-2156-10-85

**Published:** 2009-12-18

**Authors:** Kristine L Bucasas, Gagan A Pandya, Sonal Pradhan, Robert D Fleischmann, Scott N Peterson, John W Belmont

**Affiliations:** 1 Department of Immunology, Baylor College of Medicine, Houston, TX 77030, USA; 2 Pathogen Functional Genomics Resource Center, J. Craig Venter Institute, Rockville, MD 20850, USA; 3 Department of Molecular and Human Genetics, Baylor College of Medicine, Houston, TX 77030, USA

## Abstract

**Background:**

Whole genome amplification (WGA) offers new possibilities for genome-wide association studies where limited DNA samples have been collected. This study provides a realistic and high-precision assessment of WGA DNA genotyping performance from 20-year old archived serum samples using the Affymetrix Genome-Wide Human SNP Array 6.0 (SNP6.0) platform.

**Results:**

Whole-genome amplified (WGA) DNA samples from 45 archived serum replicates and 5 fresh sera paired with non-amplified genomic DNA were genotyped in duplicate. All genotyped samples passed the imposed QC thresholds for quantity and quality. In general, WGA serum DNA samples produced low call rates (45.00 +/- 2.69%), although reproducibility for successfully called markers was favorable (concordance = 95.61 +/- 4.39%). Heterozygote dropouts explained the majority (>85% in technical replicates, 50% in paired genomic/serum samples) of discordant results. Genotyping performance on WGA serum DNA samples was improved by implementation of Corrected Robust Linear Model with Maximum Likelihood Classification (CRLMM) algorithm but at the loss of many samples which failed to pass its quality threshold. Poor genotype clustering was evident in the samples that failed the CRLMM confidence threshold.

**Conclusions:**

We conclude that while it is possible to extract genomic DNA and subsequently perform whole-genome amplification from archived serum samples, WGA serum DNA did not perform well and appeared unsuitable for high-resolution genotyping on these arrays.

## Background

Array technologies are designed to rapidly genotype hundreds of thousands of single nucleotide polymorphisms (SNPs) across the genome using a relatively small amount of DNA. Advances in genotyping platforms have allowed cost-effective, whole-genome scans of multiple individuals in large-scale association studies. These studies are aimed at identifying genetic factors affecting many important complex human diseases. DNA samples from carefully characterized populations that are necessary to carry out adequately powered genome-wide association studies (GWAS), however, are often limiting. Collecting a sufficient number of appropriate samples for GWAS can be a complex and expensive collaborative challenge. There are potential alternative sources of genomic DNA for which important medical phenotypes have been recorded but their utility for GWAS has been poorly explored.

Archiving of serum samples is widely practiced in research and clinical domains [1]. Records of phenotype information associated with individual serum samples may be valuable for the GWAS setting. Archived serum samples are, therefore, an attractive and convenient potential source of genomic DNA. As an obstacle to genotyping, limited DNA yield could be overcome by taking advantage of recent whole-genome amplification (WGA) technologies [2-8]. Multiple displacement amplification (MDA), in particular, is an improved WGA technology known to minimize amplification bias, incomplete genome coverage, and generation of relatively short fragments. MDA utilizes a highly processive ϕ29 DNA polymerase and a mix of random hexamer primers and is capable of amplifying partially degraded and low quantity DNA sources. Reliable MDA-based whole-genome amplification of DNA from serum samples has been demonstrated [9]. Previous studies have successfully utilized WGA DNA from archived sera on a relatively small number of SNP markers using single assay methods [10-12]. A recent study by Mead et al showed that WGA serum DNA could be used for custom targeted medium-throughput genotyping[13]. Nevertheless, an unbiased genome wide scan with SNP markers using residual DNA in archived serum specimens will require the use of high throughput array-based genotyping. It is, therefore, necessary to determine whether this is technically feasible with standard technologies that are currently available for GWAS.

In this study, we describe a large and statistically robust study of genotyping WGA serum DNA on a widely-used whole genome SNP-genotyping panel. The Genome-Wide Human SNP Array 6.0 features about 1.8 million genetic markers, including assays for more than 906,600 single nucleotide polymorphisms (SNPs) and 946,600 probes for detection of copy number variation[14]. We further compare the performance of two different genotype-calling algorithms that are compatible with SNP 6.0 genotyping array (Birdseedv.2.0 and CRLMM). Compared to previous studies, the results of our work provide more precise and accurate estimates of genotyping efficiency and error rates using WGA serum DNA. Establishing archived serum samples as a reliable, alternative DNA source may boost the power of large-scale GWAS particularly in cases where available DNA samples from suitable subjects are limited.

## Results

### Quality control for WGA serum DNA SNP 6.0 genotyping

We successfully isolated genomic DNA from 100% of seventy-five, 20-year old 500 uL frozen serum samples using the Qiagen DNA Mini Kit (Qiagen, CA). The yield of genomic DNA ranged from 31.5 ng to 608.4 ng (mean +/- SD, 202.7 +/- 122.3 ng). Purified DNA samples were then whole-genome amplified via multiple displacement amplification (MDA). MDA was the method of choice for WGA in this study because our pilot studies using Affymetrix TG array demonstrated superior genotyping results from MDA-amplified serum DNA compared to samples amplified via other WGA methods (results not shown). Furthermore, Affymetrix recommends the use of Repli-G for their genotyping chemistry. We avoided the use of WGA methods which includes random fragmentation of DNA target because >200 bp fragment lengths must be amplified when using SNP 6.0 genotyping arrays. In this study, WGA of serum DNA samples by MDA resulted in 5500-fold to 65000-fold amplification relative to input DNA. Our yields for WGA DNA ranged from 30800 ng to 125900 ng (mean +/- SD, 18536 +/- 11754 ng). To reduce genotyping error associated directly with variation in the WGA [15,16], the 40 top-yielding samples were carried over to the genotyping process. All samples passed to the genotyping step produced relatively uniform quantity and quality of DNA (purification yield of 229 +/- 136 ng; whole-genome amplification yield of 84409 +/- 6086 ng; moderate to intense amplified PCR bands, and intact 10 kb fraction in 1% agarose gel). Two aliquots were taken from each DNA preparation for WGA and were genotyped as technical replicates. All WGA DNA were successfully run on the SNP 6.0 array, with the exception of 1 technical replicate sample. However, quality control (QC) call rates of WGA serum DNA samples ranged from 49.2% to 95.6% (mean +/- SD, 67.1 +/- 10.1%) when analyzed by the standard Birdseed algorithm[17]. WGA serum DNA performance based on the SNP6.0 QC call rates showed poor correlation with yield after DNA purification (r^2 ^= 0.122), WGA yield (r^2 ^= 0.164) and intensity of 60 bp PCR-amplified band (r^2 ^= 0.001).

### Call rates

**Figure 1:**
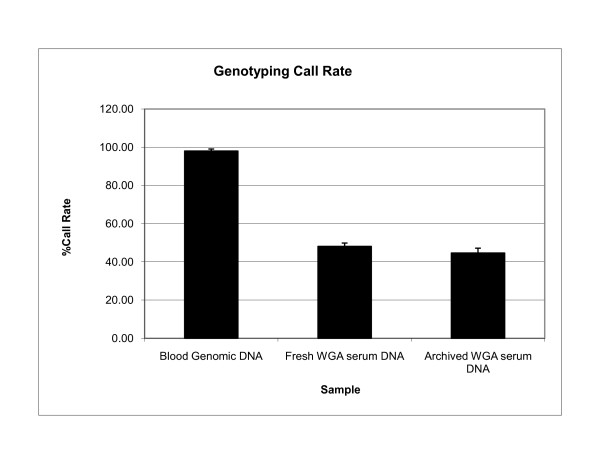
**Relative call rates between archived samples and controls**. Average genotype call rates from Birdseedv2.0 analysis at a default confidence threshold of 0.1 showed relative performance of genotyped samples on SNP 6.0 platform.

**Figure 2:**
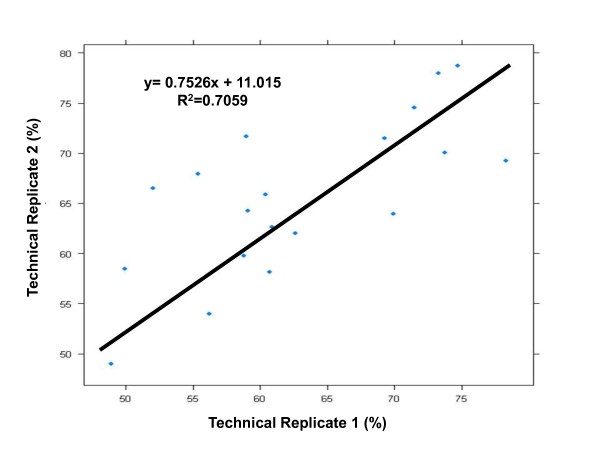
**Scattergram of call rates between serum technical replicates**. Call rates between serum technical replicates were plotted against each other to assess consistency of performance of technical replicates on the SNP 6.0 genotyping platform.

To evaluate SNP 6.0 array genotyping efficiency on WGA serum DNA samples, we measured the proportion of SNPs with missing calls in the genotyped samples. Genomic DNA from peripheral whole blood paired with whole-genome amplified DNA from freshly isolated serum of 5 individuals were run on SNP 6.0 arrays as controls. Genotype data were inferred by implementing the Birdseed v2.0 algorithm at 0.1 confidence threshold in separate clusters of non-amplified and WGA DNA samples (see Additional File 1 and Additional File 2). Percent call rates from WGA serum DNA and genomic DNA are shown in Figure 1. Overall, WGA DNA samples from sera yielded significantly low call rates (mean +/- SD, 45.0 +/- 2.7%), which corresponded to 409,366 markers out of approximately 906,600 SNP markers represented on the SNP 6.0 array. In contrast to WGA serum DNA samples, non-amplified DNA samples from peripheral whole blood yielded excellent call rates, ranging from 96.9% to 99.5% (mean +/- SD, 98.0 +/- 1.1%). The average call rate of WGA DNA samples from archived sera (mean +/- SD, 44.6 +/2.7%) did not differ from WGA DNA samples from freshly isolated sera (mean +/- SD, 48.1 +/- 1.8%) (p = 6 × 10^-5 ^). Call rates between technical replicates were correlated (r^2 ^= 0.71), thus demonstrating that high quality serum DNA samples consistently produce high genotyping call rates compared to samples with poor quality DNA(Figure 2).

### Genotype concordance between technical replicates

**Figure 3:**
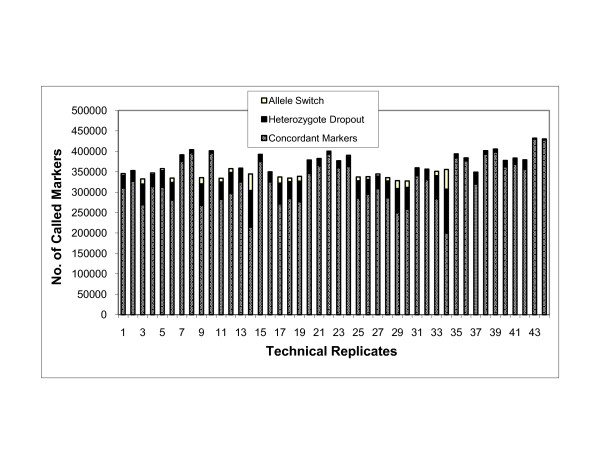
**Performance analysis of technical replicate samples**.

We further evaluated the quality of SNP 6.0 genotyping performance on WGA serum DNA samples by measuring the repeatability of calls between technical replicates as shown in Figure 3 (see Additional File 3). Reports for concordance were restricted to SNP loci with complete genotype calls on both technical replicates of a given individual. Genotype data between technical replicates gave modest percent concordances, ranging from 77.3 to 99.9 (mean +/- SD, 95.2 +/- 4.6) over approximately 340,000 SNP loci. We then determined the percent contribution of allele switch (AA ←→ BB) or heterozygote dropout (AB → AA or BB) to the observed global discordance as shown in Figure 3 (see Additional File 4). In all technical samples, discordance occurred largely due to heterozygote dropout, with an average of 85.9%. Allele switch was less common and contributed to 14.1% of the global discordance.

### Genotype concordance between paired samples

In order to assess whether accurate genotypes can be obtained from WGA serum DNA using SNP 6.0 arrays, we examined the fidelity of WGA serum DNA genotypes to their corresponding non-amplified DNA genotypes (Table 1). Calculation of concordance was restricted to SNPs where calls are made on both samples of a pair. Unlike technical replicates, paired samples showed gross discordances that ranged from 17.77% to 44.25% of the average 282,256 called markers (mean +/- SD, 31.03 +/- 12.05%). In all paired samples, the majority of discordant markers were caused by heterozygote dropout (AB→AA or BB), with an average of 50%. Of the observed heterozyogote dropouts, 69.3% were due to conversion of purine to another purine or pyrimidine to another pyrimidine. In contrast, other genotyping errors such as allele switch (AA←→BB) and heterozygote gain (AA or BB → AB) contributed an only average of 16.9% and 33.2%, respectively, to global discordance (Table 2). The genotype concordance (reproducibility) for identical samples using the SNP6.0 microarray is expected to exceed 99.9% (Affymetrix data sheet: http://www.affymetrix.com/support/technical/datasheets/genomewide_snp6_datasheet.pdf.

**Table 1:** Genotype concordance between paired samples.


**Paired Sample ID**	Total Genotype Calls*	%Concordance**	**%Discordance**

INF001	414541	55.75%	44.25%
INF002	419350	67.87%	32.13%
INF003	424260	58.86%	41.14%
INF004	453353	81.98%	18.02%
INF005	453512	82.23%	17.77%
**MEAN**	**430994**	**68.97%**	**31.03%**


*Number of markers with calls between paired samples

**%Concordance is the proportion of markers with consistent genotype calls in a paired sample.

**Table 2:** Analysis of global genotyping error in WGA serum DNA samples.


Paired Sample ID*	Allele Switch** (AA←→BB)	Heterozygote Gain**(AA or BB→AB)	Heterozygote Dropout**(AB→BB or AA)

INF001	25.8%	30.0%	44.2%
INF002	18.5%	40.5%	41.0%
INF003	23.6%	25.3%	51.1%
INF004	9.9%	32.0%	58.2%
INF005	6.6%	38.0%	55.5%
**MEAN**	**16.9**	**33.2%**	**50.0%**


*Genotype calls between a paired sample (non-amplified genomic DNA vs WGA serum DNA) were compared.

**Percentages are based on the total number of discordant markers or markers with mismatched genotype calls in paired samples

### Performance Comparison of Genotype Calling Algorithms

**Figure 4:**
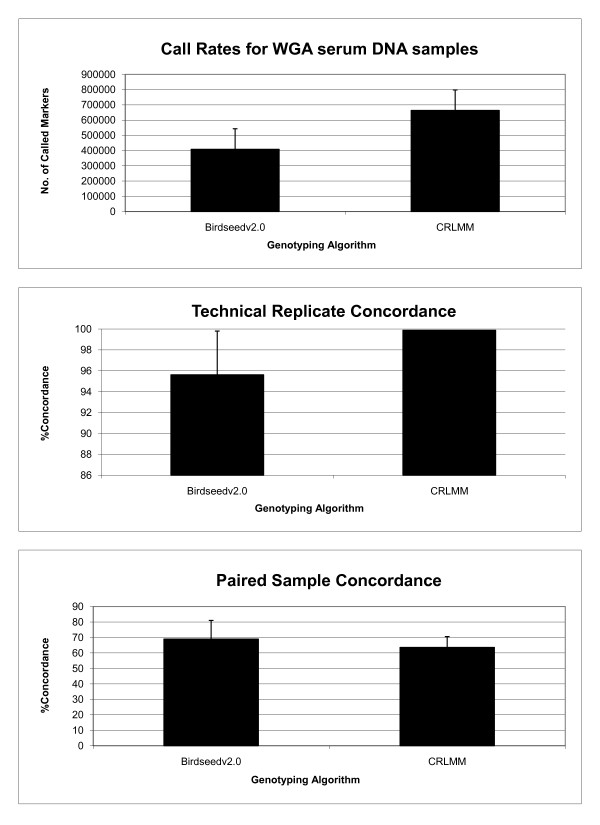
**Birdseedv2.0 vs CRLMM SNP 6.0 genotyping performance on WGA samples**. Assessment of genotype calling performance of Birdseed v2.0 and CRLMM algorithms from WGA DNA from serum samples on SNP6.0 genotyping platform is shown by comparison of (a) call rates (b) concordance between technical replicates and (c) paired sample concordance.

Previous studies have shown that genotype calling algorithms show variable performance in normalization and clustering [18]. We sought to determine whether a different clustering algorithm would improve the repeatability and accuracy of genotype inference when WGA serum DNA samples were used. Genotypes of technical replicates and paired samples were inferred using CRLMM (Corrected Robust Linear Model with Maximum Likelihood Classification) algorithm from separate signal intensity clusters of non-amplified and WGA DNA samples at 0.999 confidence threshold (see Additional File 5, Additional File 6, Additional File 7 and Additional File 8). Overall, CRLMM showed improved genotype performance in comparison to Birdseed v2.0 (Figure 4). In contrast to the Birdseedv2.0 algorithm, CRLMM yielded higher call rates for WGA serum DNA (mean +/- SD, 663320 +/- 134762 markers). Higher concordances between technical replicates were also observed, ranging from 99.77% to 99.93% (mean +/- SD, 99.87 +/- 0.001) over an average of 542,538 markers. However, CRLMM exhibited very low tolerance to poor quality samples, showing 78.75% sample rejection rate. Similar to the Birdseed v2.0, CRLMM showed gross discordance between non-amplified genomic DNA and corresponding WGA serum DNA (mean +/- SD, 36.39 +/- 6.98).

### Chip Quality Measures as Sample Rejection Determinants

**Figure 5:**
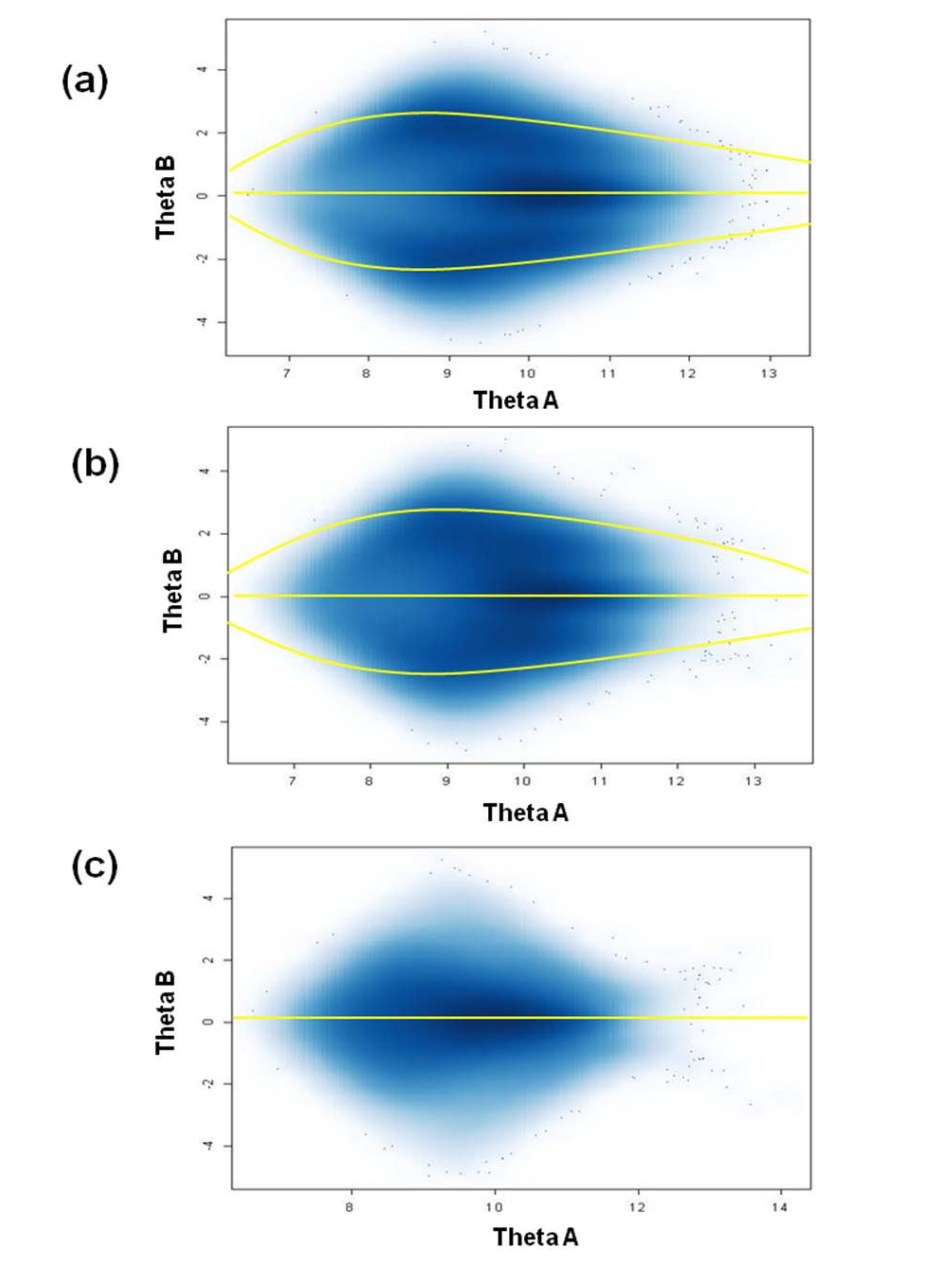
**Chip quality assessment of good performing sample vs bad performing sample on SNP 6.0 genotyping platform**. Scattergram plots of probe intensity values for allele B (Theta B) against allele A (Theta B) were generated from arrays of a (a) non-amplified genomic DNA sample, (b) good performing WGA DNA from serum and (c) bad performing WGA DNA from serum. Each point in the plot represents an assayed marker on the SNP 6.0 platform. Dark areas in the plot represent space with high-density points whereas light areas represent space with low-density points.

To investigate the basis for sample rejection by CRLMM, we visually assessed the chip quality of WGA samples. Signal intensities from probe A (Theta A) and probe B (Theta B) of SNPs assayed on SNP6.0 platform were plotted to capture the extent of differences between the intensity values AA, AB and BB genotypes. In figure 5, we show that sample performance correlated well with the separation of genotype clusters on the chip of a given sample. Genotyped samples with high confidence values and high call rates, such as genomic DNA (Figure 5a) or a representative successful WGA serum DNA (Figure 5b), showed three distinct clusters, corresponding to the three genotypes. On the other hand, very poor separation of clusters is the hallmark of the poor-performing and rejected WGA DNA samples (Figure 5c).

### Effects of Amplicon Fragment Length on Individual Marker Performance

We next determined whether amplicon fragment length affect genotype concordance and marker genotyping efficiency. As a measure of marker genotyping efficiency, we computed the average confidence values and concordance rates for each marker. Good markers were consistently called at a high confidence level across samples, and therefore produced high average confidence values regardless of amplicon fragment length (see Additional File 9). Moreover, good markers exhibited high concordance rates across all WGA serum DNA regardless of amplicon fragment length (see Additional File 10). The correlation of average confidence values against amplicon fragment length or genotype concordance was 0.007 and 0.016, respectively.

## Discussion

Our data show that DNA from archived serum samples cannot substitute for good quality genomic DNA using the standard technologies employed in this study. In our effort to establish serum as an alternative DNA source for future large-scale GWAS, we have extensively tested the performance of WGA DNA recovered from sera of 40 twenty-year old archived samples on the SNP 6.0 platform. Large standard deviations in DNA yield during the early stage of DNA preparation suggested that DNA from archived serum samples were variably prone to degradation. Selection of WGA DNA samples with uniform yield and quality did little to improve genotyping performance. Of note, all WGA DNA from serum samples in the study gave call rates of less than half of the total markers assayed on SNP 6.0 platform, far below the call rates of our non-amplified genomic DNA controls (mean +/- SD, 98.03 +/- 1.05%). More importantly, genotypes of WGA DNA from serum samples and their non-amplified DNA counterpart showed gross mismatches affecting 80-300,000 markers out of ~430,000 total called markers. For samples in which non-amplified genomic DNA were available, the relative proportion of mismatches as reflected by %discordance was consistent between paired samples (Table 1) and their corresponding technical replicates (see Additional File 3). For example, the best performing sample (INF002) consistently produced the least %discordance in both paired sample and technical replicate compared to other samples. Our data altogether suggest that the quality of DNA that can be extracted from archived serum samples is inherently very poor. This factor alone accounted for the vast majority of genotyping failure in this study.

We further studied possible sources of inaccurate genotypes. Heterozygote dropout was the most common error detected in discordant assays in both technical replicates (85%) and paired samples (50%). This suggests that DNA in archived serum samples were prone to allele bias during WGA. Allele switch in genotypes of technical replicates may have arisen from generation of amplification error during WGA stage although poor genotype calling is the more likely explanation. This accounted for <15% discordances in technical replicates and 16.9% in paired samples of the discordant genotypes. Lower than average probe signal intensities (Figure 5c), which resulted in loss of genotype information or heterozygote gain (33% discordant markers in paired samples), may have been caused by inefficient amplification during WGA due to the variable presence of highly degraded DNA fragments in the serum samples. Such errors in WGA were previously reported particularly when suboptimal DNA sources were used [2,9,12,13,19]. However, poor correlation between probe performance across samples and lengths of amplified fragments on the SNP 6.0 platform suggest that the poor genotyping results from WGA serum DNA cannot be explained simply by DNA degradation (see Additional File 9 and Additional File 10). Indeed, genotypes were called and high concordance rates were observed in both small and very large fragment lengths of good performing markers.

The low genotyping efficiency and poor reproducibility of the genotype data generated with the present protocols suggest that archived serum samples will be a problematic source of DNA for large-scale GWAS [20]. To successfully locate causative variants in the genome, high genotyping efficiency and call rates are required from the initial whole-genome screen. Genotyping error also reduces statistical power in case control studies [21-23]. The CRLMM algorithm proved superior to Birdseed in improving the call rate (663320 +/- 134762 called markers) and reproducibility between technical replicates (>99% concordance between technical replicates) at the expense of very low genotyping efficiency (6/45 samples passing confidence criteria). The CRLMM confidence measure gave a useful assessment of chip quality which was readily verified by inspection of the global genotype clustering. CRLMM, therefore, is currently the optimum genotype calling algorithm for the SNP 6.0 array available to most investigators. CRLMM has been previously shown to provide more accurate genotype calls and lower drop rates compared to other Affymetrix default algorithms (BRLMM and Birdseed) when using large datasets from non-amplified genomic DNA[18]. Our data validate and extend this finding to WGA DNA from serum samples. Genotyping algorithms apply different methods for transforming raw intensity signals from chip arrays to genotype calls. The good performance of CRLMM can be explained by the robustness of its confidence metric which appears to better reflect call accuracy relative to other genotyping algorithms. Furthermore, CRLMM not only allows measurements of call accuracy but also of chip quality. However, the improvement in reproducibility of WGA technical replicates has to be evaluated cautiously. Both Birdseedv2.0 and CRLMM produced genotypes that had poor concordance between WGA serum DNA samples and their corresponding non-amplified genomic DNA. In contrast to previous and more limited studies with targeted custom genotyping panels, our extensive survey of archived serum samples and our use of paired serum/genomic samples clearly demonstrated poor genotyping performance of WGA samples on the SNP6.0 platform. Nevertheless, our study showed that approximately 300K genotypes could be reproducibly retrieved from 20-year old sera. This suggests that with further improvements in the laboratory protocols, serum could serve as a reliable, alternative DNA source for genetic studies. Other genome-wide genotyping platforms can be explored for genotyping suboptimal samples such as serum DNA. Of these platforms, the Illumina Infinium II assay offers comparable marker density as the SNP6.0 platform[24]. Illumina Infinium II utilizes the bead technology wherein each bead contains locus specific probes, and allelic discrimination occurs through single-base primer extension reactions. In addition, several genotype-calling algorithms were developed for specific application to the Illumina assays such as GenCall[25], GenoSNP[26] and Illuminus[27]. There is a possibility of improving call rates, call accuracy and reproducibility because these platforms utilize different chemistries and genotype-calling algorithms.

## Conclusions

Archived serum samples may be useful in cases where few putative causal variants have to be replicated in an independent set of samples. In this study, we showed that procedures could be applied to maximize the quality of genotype information from WGA DNA from serum samples. We have proposed to eliminate poor quality samples in downstream data analysis by imposing QC measures based on chip quality analysis. We have also shown that genotyping algorithms such as CRLMM could better analyze genotype data from less-optimal DNA source. Moreover, analysis can be limited to genotypes with high confidence values to improve data quality. However, further investigation is still necessary to determine the appropriate criteria needed to identify poor quality samples before the genotyping stage. It is not impossible though, that marked improvement in WGA and/or genotyping platform technologies may boost the reliability of serum as an alternative, cost-effective DNA source for future association studies.

## Methods

### Sample description

DNA was purified from 40 anonymized archived sera of participants from 3 pooled influenza vaccine clinical trials performed from 1981-1986 at Baylor College of Medicine, Houston, TX. Samples were selected from 695 individuals and were categorized as high-responders and low-responders based on rise in antibody responses before and after immunization. As controls, additional DNA samples were obtained from peripheral whole blood and serum samples of 5 adult volunteer donors. Informed consent was obtained from these participants. This protocol was approved by Baylor College of Medicine's Institutional Review Board (IRB).

### DNA purification from peripheral whole blood samples

Peripheral whole blood samples were collected in 10 mL citrate dextran (ACD), yellow top BD vacutainer blood tubes (VWR International, PA). Samples were kept at room temperature and processed within 3 days after collection. Purification of genomic DNA from peripheral whole blood was carried out using Gentra Puregene Blood Kit (Qiagen, CA) according to manufacturer's protocol. DNA quantity and A260/A280 ratios were measured using Nanodrop-ND-1000 spectrophotometer (NanoDrop Technologies, Inc, DE). Purified genomic DNA were stored at 4°C until ready for use.

### Preparation of whole-genome amplified DNA from serum

Peripheral whole blood samples were collected in 5 mL BD Vacutainer red top tubes (VWR International, PA). Fractionation of serum was carried out by allowing blood samples to clot for 30-60 minutes at room temperature followed by centrifugation at 2000 rpm for 10 minutes. Serum supernatant was removed and stored in 500 uL aliquots at -80°C. Purification of genomic DNA from serum samples was carried out using QIAamp DNA Blood Mini Kit (Qiagen, CA). About 500 uL serum was incubated with Qiagen protease and equal volumes of buffer AL at 56°C for 1 hour and then loaded into QIAamp spin column. Genomic DNA was washed, dried and eluted with 20 uL buffer TE. Genomic DNA was quantified using Nanodrop ND-1000 (NanoDrop Technologies, Inc, DE). For whole-genome amplification of purified serum DNA, Repli-g Midi kit (Qiagen, CA) was used according to the manufacturer's instructions in the user-developed protocol for serum and plasma samples. For positive control, 10 ng human genomic DNA were used in every run. Briefly, 5 μL of purified serum DNA underwent denaturation step by incubating with equal volume of reconstituted buffer DLB on ice for 10 minutes. The reaction was neutralized by adding 10 μL of reconstituted stop solution. Whole genome amplifications were carried out using thermocycler (PTC-225, MJ Research Inc, MA) by incubating 30 μL DNA solution with ϕ29 DNA polmerase in 40 uL master mix, as provided by the manufacturer, at 30°C for 16 hours followed by enzyme inactivation at 65°C for 3 minutes. WGA serum DNA were quantified using Nanodrop ND-1000 (NanoDrop Technologies, Inc, DE). Samples were frozen at -20°C and genotyped within 3 weeks of whole genome amplification.

### Quality Control for WGA serum DNA samples

Starting DNA material from archived serum samples must not be highly degraded to ensure successful application in WGA and GenomeWide Human SNP Array 6.0. QC metrics were imposed at different stages of DNA preparation so that only samples with acceptable DNA quality were allowed to reach the genotyping process. Whole-genome amplification was restricted to purified DNA samples with yield ≥90 ng. Samples for whole-genome genotyping were required to have WGA yield of ≥40 ug. Samples, which passed the QC thresholds for yield, were further tested for DNA quality. To assess purity of WGA serum DNA samples, A260/280 ratios were measured using Nanodrop ND-1000 (NanoDrop Technologies, Inc, DE). The extent of DNA degradation was initially assessed in WGA serum DNA samples by carrying out 60 bp fragment amplification by PCR. Furthermore, WGA serum DNA samples were run on 1% agarose gel to detect the presence of a heavy band around the 10 kb region representing non-fragmented serum DNA. Samples with good DNA quality have A260/280 ratio>1.80, 10 kb band on 1% agarose gel and positive 60 bp fragment amplification by PCR. All genotyped samples passed all yield and quality QC requirements.

### Whole-genome genotyping

Genotyping was performed on replicate samples from the same DNA preparation using Genome-Wide Human SNP Array 6.0 Platform (Affymetrix, CA). The recommended protocol was followed as described in Affymetrix manual http://www.affymetrix.com/support/downloads/manuals/snp6_atp_userguide.pdf. Briefly, all DNA samples were normalized to 50 ng/μL. Two 5 _L (250 ng) aliquots were made from each DNA sample followed by digestion with either NspI or StyI restriction enzymes. Biotin-labeling primer amplification assay was performed on the resulting DNA fragments in each aliquot. The amplified products were fragmented, combined and purified using polystyrene beads. Samples were injected into the cartridges housing the oligonucleotide arrays, hybridized, washed and stained. The washing and staining procedures were run on Affymetrix fluidics station 450. Mapping array images were obtain by scanning with GeneChip Scanner 3000 7G (Affymetrix, CA). CEL files containing raw signal intensities were stored and used for downstream analysis of WGA serum DNA performance on Genome-Wide Human SNP Array 6.0 Platform (Affymetrix, CA) as described below.

### Data Analysis Scheme

#### Datasets

A total of 100 CEL files were generated from 50 sample replicates, which consisted of 40 WGA-DNA from archived serum samples and 5 WGA-DNA from serum samples paired with 5 genomic DNA from peripheral blood. WGA serum DNA and whole blood genomic DNA CEL files were clustered separately during genotype calling as recommended in Affymetrix user-developed protocol for genotyping WGA-DNA samples. Cluster files for WGA serum DNA consisted of 89 CEL files. CEL files from 270 HapMap samples were obtained from Affymetrix and were used to cluster with whole blood genomic DNA CEL files to improve the accuracy of genotype calls.

#### Inference of Genotype Calls

Genotype calls were inferred using two recently available, SNP6.0-compatible algorithms based on raw intensity signals stored in CEL files: (1) Birdseedv2.0 [17,28] and (2) CRLMM (Corrected Robust Linear Model with Maximum Likelihood Classification) [29,30]. The algorithms selected for genotype calling differ in methods for estimating the boundaries of each genotype clusters and for assigning genotype calls. Birdseedv2.0 was implemented using the Affymetrix, CA Power Tool version 1.8.5 (apt-1.8.5) as supported in Affymetrix, CA website http://www.affymetrix.com/support/developer/powertools/apt_archive.affx. For genotype calling, Birdseedv2.0 preprocessed the probe signal intensities across all chips based on BRLMM[31,32]. Expectation Maximization (EM) algorithm was used to fit a Gaussian mixture models in 2-dimensional signal A and signal B space. A pseudo-Bayesian approach was applied to ensure that the boundaries derived for each genotype cluster are very close to the supplied SNP-models. Output genotype calls were coded as 0, 1, 2, and -1 for AA, AB, BB and no signal, respectively. Confidence measures were reported as a measure of certainty genotype prediction based on the fitted models for each genotype. For genotype calling using CRLMM algorithm, Oligo package v1.2.2 was downloaded (Personal Communication, Benilton Carvalho) and run in R statistical software (v2.6.1). Raw signal intensities for each probe were normalized similar to Robust Multi-array Average (RMA) after correction of sequence and fragment length effects on log intensities. To further enhance the precision of genotype inference, gender information for each sample was supplied as a covariate in a separate phenoData object. Log normalized intensities for SNP alleles were recorded and used to estimate the genotype regions, which served as a basis for classifying genotype calls. A likelihood based distance function was applied to estimate the genotype calls and corresponding confidence values. Genotypes in the output matrix were coded as 1, 2, 3 to represent AA, AB, BB calls, respectively. A separate matrix containing confidence values were reported to give an estimate of certainty for each given call. Genotype calls using Birdseedv2.0 and CRLMM were inferred at the default confidence thresholds of 0.1 and 0.999, respectively.

#### Performance Analysis

Output files were uploaded to R statistical software package (v2.6.1). Per-sample %call rates and concordance between replicate or paired DNA samples were calculated to measure the performance of genotyped samples. The quality of genotyped sample was measured by the extent of genotype call dropouts. Call rate is defined as the number of SNPs with successfully called genotype out of total number of assayed SNP markers. Robustness and repeatability of genotype calls were measured by concordance between replicate and paired samples. Concordance is defined as the number of SNPs with consistent calls between replicate or paired samples. Blood genomic DNA was set as the gold standard for determining the accuracy of calls derived from WGA serum DNA samples. Performances of CRLMM and Birdseedv2.0 genotype calling algorithms when using WGA serum DNA were determined by comparing percent call rates and concordance results. An optimum genotype calling algorithm for WGA serum DNA sample should be able to distinguish good performing and bad performing samples while maximizing call rates and concordance between paired and replicate samples.

#### Reproducibility

Inconsistencies in calls between replicate samples may result from errors in whole genome amplification process due to allele bias amplification or inaccurate definition of genotype clusters during genotyping. Genotype calls from Birdseedv2.0 were uploaded in R and Microsoft Access. SNPs with discordant calls between replicate WGA serum DNA samples were tallied. Of the total discordant calls, the proportion of SNPs with heterozygote dropout (AB->AA or BB) or allele switch (AA->BB) conversion were recorded and compared. If discordance between replicate samples were random, equal occurrence of heterozygote to homozygote conversion and allele 1 homozygote to allele 2 homozygote conversion between replicate samples should be expected. Marked increase in heterozygote to homozygote conversion may suggest allele bias amplification during whole-genome amplification of serum DNA. On the other hand, increased frequency of allele 1 homozygote to allele 2 homozygote calls may reflect errors in defining genotype clusters during genotype calling.

#### Relationship of Fragment Length and Reproducibility to SNP Performance

Performance of individual SNPs may be affected by the extent of degradation in the starting serum DNA or difficulty in amplification of large DNA fragment during whole genome amplification process. Effect of varying fragment lengths on SNP performance was evaluated. Information on NspI or StyI fragment size associated with the assayed SNP was extracted from Genome-Wide Human SNP Array 6.0 annotation. Using Birdseedv2.0 output files, SNP performance was measured by calculating the average confidence values for each SNP across all genotyped WGA serum DNA samples. SNPs with low average confidence values are poor performers whereas SNPs with consistently high confidence values are good performers. SNP performance was also measured by calculating genotype concordance between technical replicates. SNPs with low average concordance rates are poor performers whereas SNPs with consistently high average concordance rates are good performers. Linear regression was performed on average confidence values against fragment size using R statistical software. The relationship between repeatability of genotype calls between replicate samples and SNP performance was also evaluated. Linear regression was performed on average confidence values against concordance between replicate samples to identify correlation using R statistical software.

#### Assessment of Signal Quality

Dropouts in calls or inconsistencies between replicates calls may result from non-distinct separation of clusters for each genotype. Quality of signal intensities for good and bad performing WGA serum DNA samples was visually examined. Normalized log signal intensities for probe A (θ_A _) and probe B(θ_B _) for each SNP were extracted from CRLMM output files using R statistical package software (v2.6.1)[30]. Geneplotter package was used to generate 2D density plots of θ_B _against signal θ_A _. Signal to noise ratio was assessed by examining the stratification of estimated regions for homozygous and heterozygous genotype calls. Good quality samples were expected to display distinct boundaries for each genotype cluster.

## Authors' contributions

This study was developed and designed by KLB and JWB. KLB carried out sample preparation, implemented genotype calling algorithms and performed data analysis. SNP, GAP, SP and RDF coordinated and carried out SNP 6.0 genotyping of serum samples. KLB, GP, SNP and JWB contributed to the writing of the paper. All the authors have read and approved the final manuscript.

## Supplementary Material

Additional file 1 Birdseed non-WGA DNA raw genotype output. Click here for file

Additional file 2 Birdseed serum WGA DNA raw genotype output. Click here for file

Additional file 3 Raw concordance analysis of technical replicate samples. Click here for file

Additional file 4 Raw global discordance analysis between technical replicates. Click here for file

Additional file 5 CRLMM non-WGA DNA raw genotype output. Click here for file

Additional file 6 CRLMM serum WGA DNA raw genotype output. Click here for file

Additional file 7 CRLMM serum WGA DNA genotype confidence. Click here for file

Additional file 8 CRLMM serum WGA DNA genotype confidence. Click here for file

Additional file 9 Scatter Plot of Probe Mean Confidence Value against Fragment Length. Click here for file

Additional file 10 Scatter Plot of Probe Mean Concordance against Fragment Length. Click here for file
