# Supplementary figures and images for: Assessing the utility of whole-genome amplified serum DNA for array-based high throughput genotyping
Source: BMC Genet. 2009 Dec 18;10:85. doi: 10.1186/1471-2156-10-85 (PMC2803178; doi:10.1186/1471-2156-10-85)

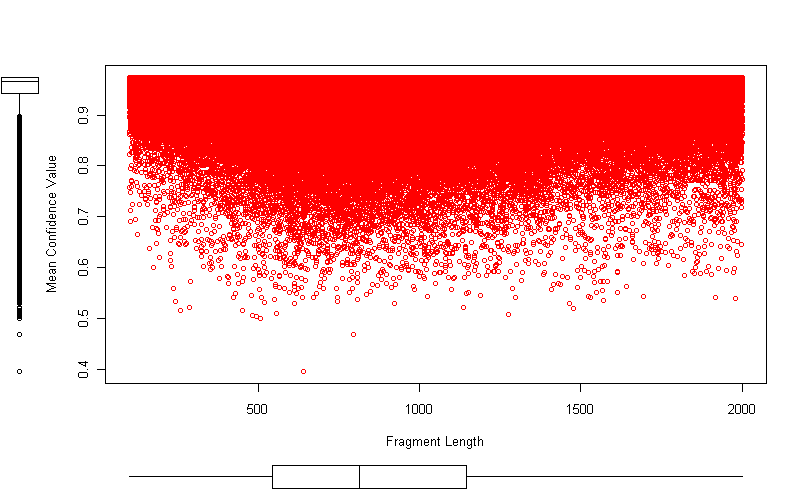

Supplement: Additional file 9 — Scatter Plot of Probe Mean Confidence Value against Fragment Length. [file 1471-2156-10-85-S9.TIFF]

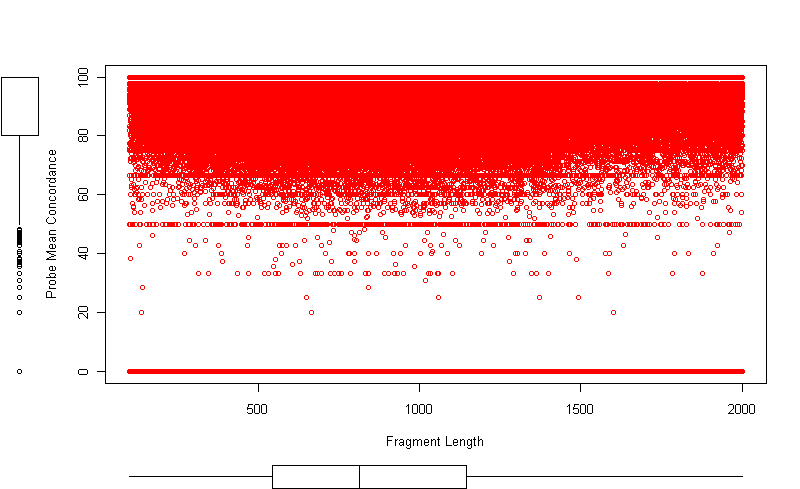

Supplement: Additional file 10 — Scatter Plot of Probe Mean Concordance against Fragment Length. [file 1471-2156-10-85-S10.TIFF]
